# Effects and underlying mechanisms of irisin on the proliferation and apoptosis of pancreatic β cells

**DOI:** 10.1371/journal.pone.0175498

**Published:** 2017-04-10

**Authors:** Shiwei Liu, Fang Du, Xin Li, Mingming Wang, Ruixue Duan, Jiaxin Zhang, Yaru Wu, Qi Zhang

**Affiliations:** 1 Department of Endocrinology, Shanxi DAYI Hospital, Shanxi Medical University, Taiyuan, China; 2 Graduate School of Shanxi Medical University, Taiyuan, China; University of Hull, UNITED KINGDOM

## Abstract

Pancreatic β cell dysfunction and reduction due to glucose toxicity play a crucial role in the development of type 2 diabetes mellitus (T2DM). Irisin, a novel exercise-induced myokine, reduces obesity, improves insulin resistance and lowers blood glucose by promoting the browning of white adipose tissue, thereby enhancing thermogenesis and increasing energy expenditure. Recent studies have reported that irisin promotes cell proliferation and protects cells from apoptosis. However, the effects of irisin on pancreatic β cells are unknown. Thus, the aim of this study was to investigate the effects and the potential underlying mechanisms of irisin on pancreatic β cell proliferation and apoptosis induced by high glucose. Both in vitro (INS-1 cells) and in vivo (a T2DM rat model) experiments were conducted. Irisin significantly increased the proliferation of INS-1 cells, with the most significant effect observed at 24 h with 100 ng/ml irisin. Irisin also promoted INS-1 cell proliferation via the ERK and p38 MAPK signaling pathways, protected the cells from high-glucose-induced apoptosis by regulating the expression of caspases, Bad, Bax, Bcl-2 and Bcl-xl, and improved pancreatic β cell function. Irisin significantly reduced the body weight and blood glucose values and increased the serum insulin levels of the diabetic rats. An oral glucose tolerance test (OGTT) indicated that irisin also improved the glucose tolerance of T2DM rats. Together, these findings suggest that irisin may have applications in the prevention and treatment of T2DM because of its protective effect on the secretion of pancreatic β cells.

## Introduction

Diabetes mellitus is one of the most prevalent chronic non-communicable diseases in humans worldwide. Type 2 diabetes mellitus (T2DM) patients account for the vast majority of diabetes cases. Insulin resistance (IR) and pancreatic β cell dysfunction are two major pathophysiological characteristics of T2DM. Thus, pancreatic β cells play a crucial role in T2DM pathogenesis. In the early stage of T2DM development, the pancreas first increases insulin release by improving the function of β cells or increasing their number when IR occurs in peripheral tissues. However, β cell apoptosis caused by high-glucose toxicity results in decreased β cell function and insulin sensitivity, further reducing insulin secretion in diabetic patients with long-term hyperglycemia. Consequently, a vicious cycle forms if high glucose is not properly controlled. The body shows only impaired glucose tolerance at first, but eventually, T2DM develops when the number of pancreatic β cells decreases to a certain extent and when the increase of insulin can no longer compensate for IR. Therefore, maintaining a certain number of pancreatic β cells is essential to ensure sufficient compensation for IR.

Irisin, a newly discovered myokine, is an adipose cytokine predominantly produced from fibronectin type III domain-containing protein 5 (FNDC5) of skeletal muscle in response to exercise in both mice and humans[1,2]. This molecule increases uncoupling protein 1 (UCP1) and browning of white adipose tissues, thereby enhancing thermogenesis and energy consumption of the adipose tissue[1]. Moreover, it can improve insulin resistance, lower blood glucose and promote weight loss. Studies have shown that irisin also promotes cell proliferation and inhibits cell apoptosis. Some researchers have reported that irisin can enhance the proliferation of human umbilical vein endothelial cells and maintain their levels[3]. Irisin was also reported to increase the proliferation of H19-7 mouse hippocampal neurons[4]. Meanwhile, irisin can inhibit the high-glucose-induced apoptosis of vascular endothelial cells and improve their function through the ERK and AMPK-PI3K-Akt-eNOS signaling pathways[3,5,6]. Irisin has also been shown to protect against palmitic acid-induced apoptosis in liver cells by inhibiting oxidative stress and inflammation[7].

However, it is unclear how irisin affects pancreatic β cells. Therefore, the aim of this study was to investigate the effects and underlying mechanisms of irisin on pancreatic β cell proliferation and apoptosis.

## Materials and methods

### Cell culture

Rat pancreatic β cells (INS-1 cells) were purchased from the American Type Culture Collection (ATCC; Manassas, VA, USA). The cells were cultured in RPMI 1640 medium supplemented with 15% fetal calf serum, 100 U/ml penicillin, and 100 μg/ml streptomycin at 37°C in a humidified atmosphere incubator with 5% CO_2_. The culture medium was replaced every 2 days, and the cells were passaged when their density reached 80–90%. The INS-1 cells were treated with high glucose and recombinant irisin protein (Phoenix Pharmaceuticals, Belmont, CA, USA).

### Cell proliferation assay

INS-1 cell proliferation was measured using the Cell Counting Kit-8 (CCK-8) (Dojindo, Japan). Cells were seeded into a 96-well plate at a density of 2×10^4^ cells/well. The cells were treated with various concentrations of irisin (0, 40, 60, 80, 100 and 150 ng/ml) or with 100 ng/ml irisin for different lengths of time (6, 12, 18, 24, and 48 h). In each experiment, a blank and the negative control group were included. A volume of 10 μl of CCK-8 solution was added to each well before incubation was completed. The viability of the cells was determined using a spectrophotometer plate reader at an absorbance of 450 nm.

### Analysis of apoptosis

INS-1 cell apoptosis was measured with the annexin V-FITC/propidium iodide (PI) bi-label assay. INS-1 cells were seeded into 6-well plates at 5×10^5^/well in RPMI 1640 containing 10% FBS, 10 ng/ml bFGF and 10 ng/ml EGF. After starvation in serum-free media for 24 h, the cells were pretreated with 25 mmol/l glucose for 24 h and incubated with or without 100 ng/ml irisin for another 24 h. The cells were then harvested, washed and resuspended in cold phosphate-buffered saline (PBS). Apoptotic cells were identified with an annexin V-FITC apoptosis detection kit (BD Biosciences, USA) according to the manufacturer’s protocol. Briefly, the cells were washed and subsequently incubated for 15 min at room temperature in the dark with 100 ml of 1× binding buffer containing 5 μl annexin V-FITC and 10 μl PI. The apoptotic rate was determined using a BD Accuri C6 flow cytometer and processed using FlowJo (Ashland, OR, USA) software.

### Animals

Thirty male Sprague Dawley rats (200–220 g) were purchased from Beijing Vital River Laboratory Animal Technology Co., Ltd. The rats were housed in the Laboratory Animal Center of Shanxi Medical University. All animals were maintained in 22–26°C, 40–60% humidity and a 12-h light/dark cycle, and they were given food and water ad libitum. The use of experimental animals was in strict accordance with the Regulations for the Administration of Affairs Concerning Experimental Animals, and the study protocol was approved by the Institutional Animal Care and Use Committee of Shanxi Medical University.

### Induction of diabetes and treatment with irisin

After one week of adaptive feeding, the rats were randomly assigned to a normal diet group (NC group, n = 10) and a high-fat diet group (HFD group, n = 20). The normal diet contained 57% carbohydrate, 18% protein, and 25% fat; the high-fat diet contained 37% carbohydrate, 13% protein and 50% fat. After 16 weeks of dietary manipulation, HFD-fed rats were given a single dose of streptozotocin (STZ) (30 mg/kg, i.p.) to induce diabetes. Rats with fasting blood glucose (FBG)≥11.1 mmol/l were considered diabetic and selected for the study. The T2DM rats were randomly divided into a diabetic group (T2DM group, n = 8) and an irisin treatment group (Irisin group, n = 8). The irisin group was treated with irisin at doses of 100 ng/ml intraperitoneally for 8 weeks. The rats in the NC group and T2DM group were injected with a corresponding volume of normal saline on the same schedule. The dose of irisin was selected based on previous cell culture studies. The body weight and FBG of the rats were measured at the end of the intervention. The animals were observed twice per day for morbidity and mortality from the beginning of the study until termination. All animals survived the study period without observable clinical signs other than symptoms related to T2DM; therefore, no pain reduction method was used. At the end of the study, the rats were euthanized with intraperitoneal injection of sodium pentobarbital (50 mg/kg BW).

### FBG and serum insulin levels

FBG and serum insulin were assessed in overnight-fasted rats at the end of the intervention. Blood samples were obtained from the tail vein, and glucose concentrations were evaluated using a blood glucose meter (Johnson & Johnson, New Jersey, USA). Serum insulin levels were measured using an ELISA kit (Jianglaibio, Shanghai, China) following the manufacturer’s instructions at an optical density of 450 nm using a microplate reader (Bio-Rad Laboratories, Inc., USA).

### Oral glucose tolerance test (OGTT)

At the end of the intervention, an OGTT was performed in overnight-fasted rats. Each experimental animal received a single dose of 1.5 g of 50% glucose solution/kg body weight via gavage. Blood samples were obtained from the tail vein, and glucose values were measured with the glucose meter before glucose loading (t = 0) and 30, 60, and 120 min after glucose administration. At the same time, approximately 100 μl blood was sampled from the tail vein for insulin measurement using an ELISA kit (Jianglaibio, Shanghai, China). The area under the curve for glucose (AUC glucose) was used to assess the functionality of pancreatic β cells.

### Western blot analysis

Western blotting was used to measure the protein levels of Ki67, ERK, p-ERK, p38 MAPK, p-p38 MAPK, cleaved caspase-3, cleaved caspase-9, Bad, Bax, Bcl-2 and Bcl-xl in INS-1 cells. Briefly, irisin-pretreated cells were rinsed three times with ice-cold PBS and lysed with ice-cold lysis buffer (Sciencells 8006). The cell lysates were centrifuged at 14,000 rpm and 4°C for 15 min. The supernatant was then quantified for protein concentration using the BCA method (Bio-Rad, Hercules, USA). Equal amounts of proteins were used for sodium dodecyl sulfate polyacrylamide gel electrophoresis (SDS-PAGE) and then transferred to a polyvinylidene fluoride (PVDF) membrane. After the membrane was blocked with 5% skim milk, it was incubated with the primary antibody at room temperature for 2 h followed by horseradish peroxidase (HRP)-conjugated secondary antibody at room temperature for an additional 1 h. After three washes with PBS, the blots were visualized with an enhanced chemiluminescence (ECL) Western blot detection system (Genmed GMS30026.3). β-actin was used as a reference. The quantitative data from the Western blot were analyzed using Gel-Pro analyzer software.

### Glucose-stimulated insulin secretion

INS-1 cells were incubated for 1 h in glucose-free Krebs-Ringer bicarbonate HEPES buffer, which was supplemented with 0.1% albumin. Subsequently, the cells were incubated for 1 h in Krebs-Ringer solution containing 16.7 mmol/l glucose. The secreted insulin in the supernatant was measured by ELISA (Mercodia, Uppsala, Sweden). Protein content was determined using a Bradford protein assay.

### Statistical analysis

The results are presented as the mean ± SD, and each sample was measured in triplicate. Each experiment was repeated at least three times unless otherwise indicated. One-way analysis of variance followed by a least significant difference (LSD) test was used to calculate differences between the various study groups. A level of *p*<0.05 was considered statistically significant.

## Results

### Effect of irisin on INS-1 cell proliferation

**Fig 1:**
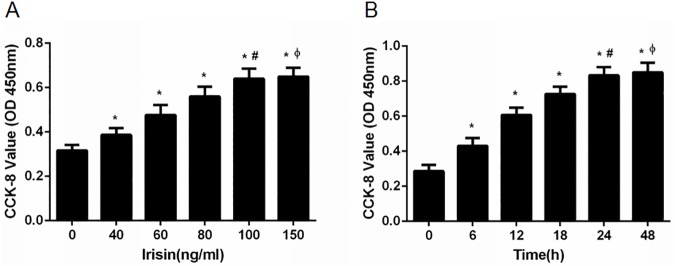
Irisin promoted the proliferation of pancreatic β cells. (A) INS-1 cells were cultured and treated with different concentrations of irisin (0, 40, 60, 80, 100 and 150 ng/ml) for 24 h. * *P*<0.05 compared with the control (0 ng/ml) group; # *P*<0.05 compared with the 40, 60, 80 ng/ml irisin-treated group; Φ *P*>0.05 compared with the 100 ng/ml irisin-treated group. (B) INS-1 cells were treated with 100 ng/ml irisin for various time intervals (0, 6, 12, 18, 24, and 48 h). * *P*<0.05 compared with 0 h; # *P*<0.05 compared with 6, 12, 18 h; Φ *P*>0.05 compared with 24 h.

**Fig 2:**
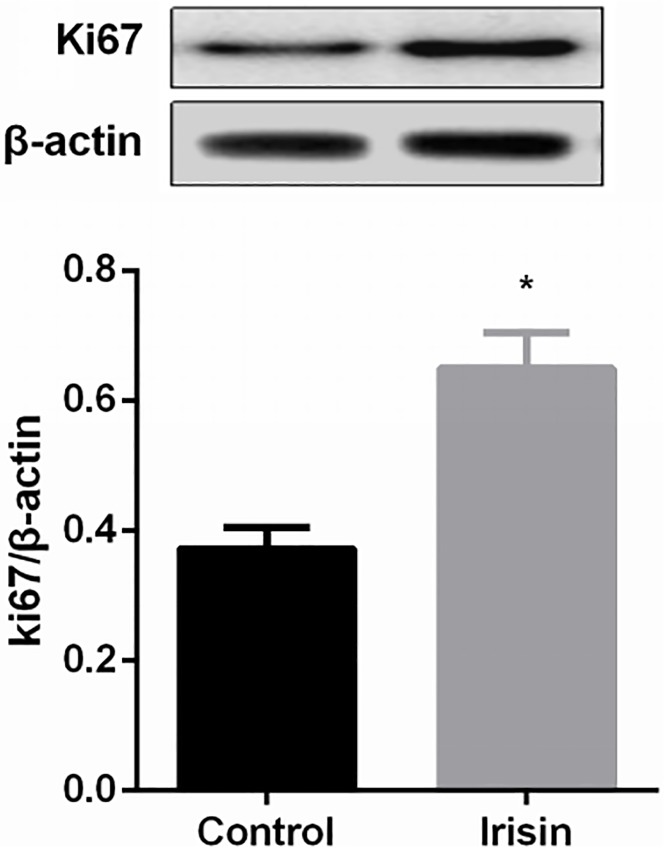
Irisin increased the protein level of Ki67. * *P*<0.05 compared with the control (0 ng/ml) group.

We used CCK-8 assays to determine whether irisin could promote the proliferation of INS-1 cells. INS-1 cells were treated with different concentrations of irisin (0, 40, 60, 80, 100 and 150 ng/ml) for 24 h. OD values of INS-1 cells treated with irisin were higher than those of cells without irisin treatment. A concentration of 100 ng/ml irisin had the greatest effect on proliferation (Fig 1A). When INS-1 cells were treated with 100 ng/ml irisin for different time intervals (0, 6, 12, 18, 24, or 48 h), the OD values were higher in the 24 h and 48 h groups than those of the other times evaluated. INS-1 cell proliferation was not significantly different between 100 ng/ml irisin for 24 h and the same concentration for 48 h (Fig 1B). Meanwhile, the protein level of Ki67, a cellular marker of proliferation, was increased significantly in the irisin-treated group (Fig 2).

### Irisin decreases body weight and FBG levels and improves insulin secretion of type 2 diabetic rats

**Fig 3:**
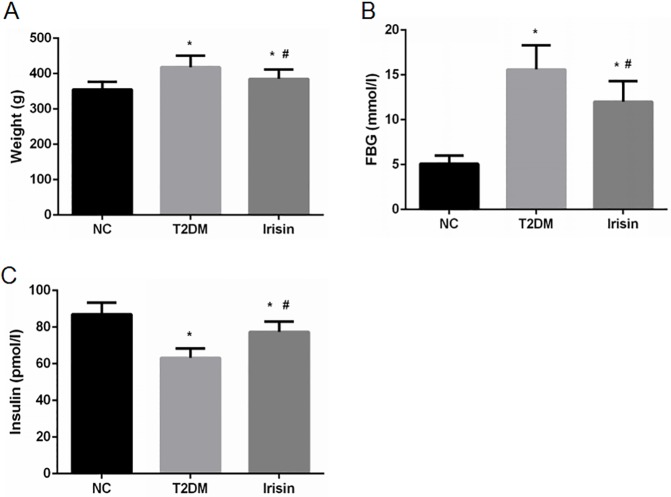
Irisin reduced the body weight and blood glucose levels and improved the insulin secretion of T2DM rats. After intervention with irisin, the body weight, FBG and serum insulin levels of the rats were determined. (A-C) The effects of irisin on body weight, FBG, and serum insulin levels, respectively. *P<0.05 compared with the NC group; # *P*<0.05 compared with the T2DM group.

The effects of irisin were also examined in vivo using a T2DM model of SD male rats. Irisin treatment significantly reduced the body weight (Fig 3A) and blood glucose levels (Fig 3B) and increased the insulin levels of type 2 diabetic rats (Fig 3C).

### Irisin improves the glucose tolerance of T2DM rats

**Fig 4:**
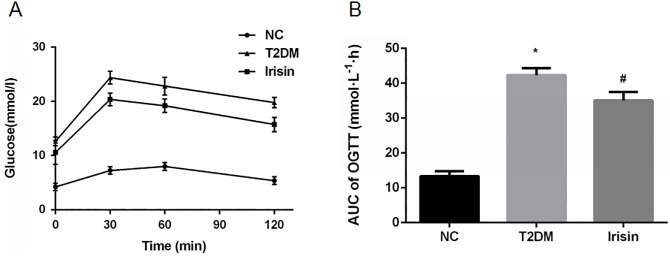
Irisin improved glucose tolerance. At the end of the intervention with irisin, an OGTT was performed in overnight-fasted rats. (A) OGTT. (B) Area under curve of OGTT. *P<0.05 compared with the NC group; # *P*<0.05 compared with the T2DM group.

At the end of the intervention, an OGTT was performed to evaluate the effect of irisin on glucose tolerance. Irisin improved glucose tolerance, as shown by a significantly decreased plasma glucose level (Fig 4A) and area under the OGTT curve (Fig 4B) in the T2DM rats treated with irisin compared with that of the untreated rats.

### Irisin promotes INS-1 cell proliferation via the ERK and p38 MAPK signaling pathways

**Fig 5:**
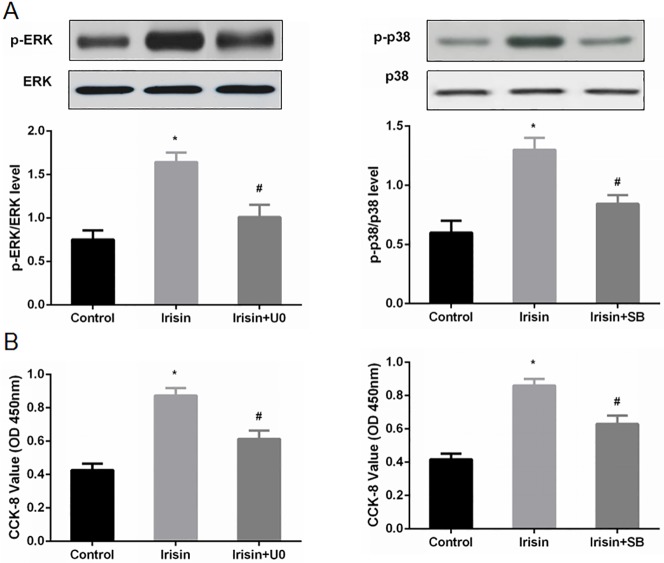
Irisin promoted pancreatic β cell proliferation via the ERK and p38 MAPK signaling pathways. INS-1 cells were pretreated with PBS (as a vehicle control), U0 (U0126, 10 μM for 30 min) or SB (SB203580, 10 μM for 30 min), and then, the cells were cultured and treated with irisin (100 ng/ml) or vehicle. (A) The protein levels of total ERK, p-ERK, total p38 and p-p38 were determined by Western blot analysis. (B) The data are expressed as OD values at 450 nm and are reported as the mean ± SD. * *P*<0.05 compared with the control. # *P*<0.05 compared with the irisin-treated group.

To further elucidate the potential mechanisms underlying irisin-induced proliferation of INS-1 cells, we investigated the protein levels of ERK and p38 MAPK, which are critically involved in cell proliferation. Significant increases in phosphorylated ERK (p-ERK) and phosphorylated p38 (p-p38) in INS-1 cells were detected by Western blotting after treatment with irisin, while the total levels of ERK and p38 were unaffected (Fig 5A).

We used the ERK inhibitor U0126 (U0) and the p38 inhibitor SB203580 (SB) to verify the roles of the ERK and p38 signaling pathways in the pro-proliferative effect of irisin. INS-1 cells were pretreated with high glucose (control), U0, or SB for 30 min and cultured in the presence of irisin. ERK and p38 phosphorylation levels were then detected. Following addition of U0 and SB, the levels of p-ERK and p-p38 were suppressed, whereas the total levels of ERK and p38 remained unchanged (Fig 5A). The CCK-8 OD values at 450 nm were decreased in INS-1 cells treated with U0 or SB (Fig 5B).

### Irisin attenuates apoptosis and improves glucose-stimulated insulin secretion from INS-1 cells

**Fig 6:**
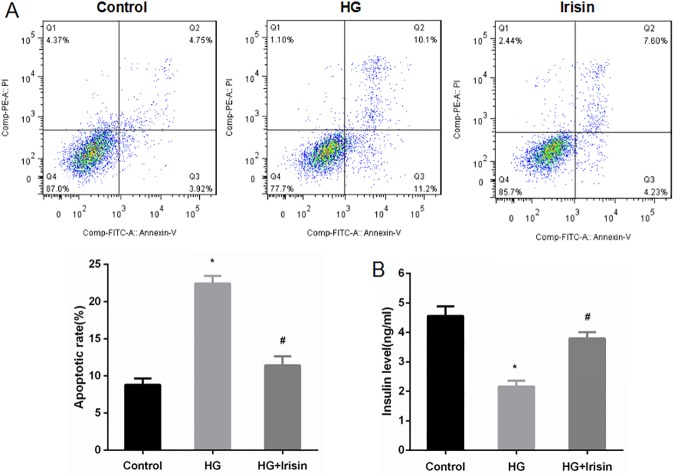
Irisin attenuated apoptosis and improved the insulin secretion induced by high glucose in INS-1 cells. (A) INS-1 cells were pretreated with PBS (control) or high glucose (25 mmol/l) for 24 h and then incubated with or without 100 ng/ml irisin for another 24 h. Apoptosis of INS-1 cells was detected by annexin V-FITC/PI staining. (B) The insulin levels were detected from cell-free supernatant after incubation with glucose at 16.7 mmol/l for 1 h. The data are expressed as the mean ± SD. * *P*<0.05 compared with the control. # *P*<0.05 compared with the high-glucose group.

INS-1 cells were pretreated with PBS as a control or high glucose at 25 mmol/l for 24 h. The cells were then treated or not treated with 100 ng/ml irisin for 24 h. INS-1 cells apoptosis was detected by annexin V-FITC/PI staining and flow cytometry. The apoptotic rate of high-glucose-treated INS-1 cells was significantly higher than that of the control group, while the irisin treatment group showed reduced apoptosis of INS-1 cells induced by high glucose (Fig 6A).

Insulin levels were detected in cell-free supernatant after the cells were incubated for 1 h in Krebs-Ringer solution, which contains 16.7 mmol/l glucose. Insulin secretion in response to stimulation with glucose was significantly reduced **i**n INS-1 cells pretreated with high glucose. Irisin treatment improved the insulin secretion of INS-1 cells stimulated with high glucose; cells treated with irisin and high glucose showed significantly higher insulin secretion than that of cells treated only with high glucose (Fig 6B).

### Irisin modulates apoptosis-related gene expression in INS-1 cells under high-glucose conditions

**Fig 7:**
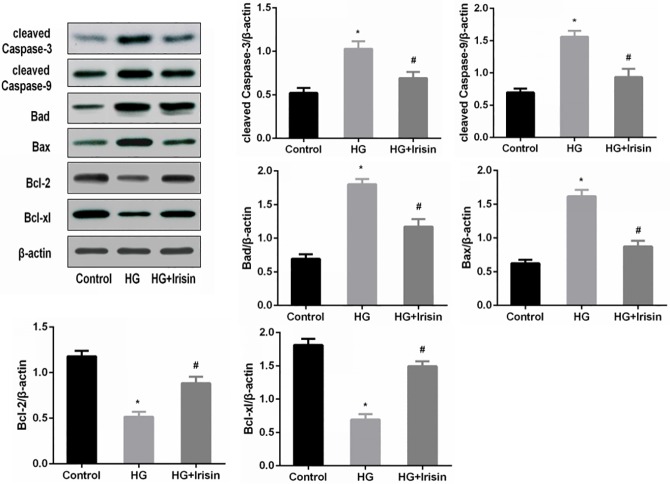
Irisin modulated apoptosis-related gene expression and protein levels in INS-1 cells. INS-1 cells were pretreated with PBS (control) or 25 mmol/l glucose for 24 h and incubated with or without 100 ng/ml irisin for 24 h. The apoptosis-related proteins cleaved caspase-3, cleaved caspase-9, Bad, Bax, Bcl-2 and Bcl-xl were detected by Western blotting. The data are reported as the mean ± SD. * *P*<0.05 compared with the control. # *P*<0.05 compared with the high-glucose group.

To further investigate the potential mechanisms by which irisin affects the apoptosis of high-glucose-treated INS-1 cells, we measured the expression levels of cleaved caspase-3, cleaved caspase-9, Bad, Bax, Bcl-2, Bcl-xl and several other key apoptosis regulatory proteins. The Western blotting results indicated that following treatment with 100 ng/ml irisin, the levels of the pro-apoptotic proteins cleaved caspase-3, cleaved caspase-9, Bad and Bax were decreased, while the anti-apoptotic proteins Bcl-2 and Bcl-xl were increased (Fig 7).

## Discussion

The dysfunction and reduction in pancreatic β cells are important causes of persistent hyperglycemia in patients with T2DM. Recent studies have demonstrated that irisin can promote cell proliferation and protect cells from high-glucose-induced damage[3]. However, the effects of irisin on pancreatic β cells are still unclear. Therefore, the aim of this study was to investigate the effects of irisin on the proliferation and apoptosis of pancreatic β cells treated with high glucose and explore the potential mechanisms of these effects.

First, INS-1 rat pancreatic cells were treated with different concentrations of irisin for 24 h, and the proliferation rates were examined. Irisin induced a dose-dependent increase in cell proliferation after 24 h, with 100 ng/ml producing the greatest effect. Further increasing the concentration of irisin to 150 ng/ml did not increase the proliferation rate, suggesting that the irisin receptors were saturated in pancreatic β cells treated with higher concentrations of irisin. However, the irisin receptor remains to be identified. The optimal irisin concentration is different between cell lines; thus, it may vary depending on the specific cell line[3,5].

We also explored the optimal duration of irisin exposure. The results showed that after 24 h, irisin at 100 ng/ml induced the highest cell proliferation. There was no significant difference between 48 h and 24 h. Thus, we selected 100 ng/ml irisin to treat INS-1 cells for 24 h in subsequent experiments.

Ki67, a cell proliferation antigen, is widely used as a marker for cellular proliferation[8]. In the current study, the protein level of Ki67 was determined in cells treated with or without 100 ng/ml irisin for 24 h. The protein level of Ki67 was increased significantly in cells treated with irisin, demonstrating that irisin can promote cell proliferation.

Subsequently, we established a type 2 diabetic rat model and then treated the diabetic rats with irisin at 100 ng/ml for 8 weeks. Irisin significantly reduced the body weight and FBG level of the rats and increased their serum insulin level. The OGTT indicated that irisin also improved the glucose tolerance of T2DM rats. Therefore, in addition to increasing energy consumption, irisin may also play an important role in glucose metabolism. Thus, irisin may regulate the number of pancreatic β cells and improve the function of these cells, which would then reduce blood glucose levels and improve glucose tolerance.

The ERK and p38 MAPK signaling pathways play an important role in cell proliferation[9,10]. Studies have indicated that irisin promotes the browning of white adipose tissue[1] and the proliferation of bone cells through the ERK and p38 MAPK signaling pathways[11]. Irisin can also increase the migration and lumen formation of endothelial cells and promote the angiogenesis of human umbilical vein endothelial cells by the ERK signal transduction pathway[12]. Therefore, this study examined whether irisin promoted the proliferation of pancreatic β cells through these pathways. After 24 h of intervention with 100 ng/ml irisin, the levels of ERK and p38 phosphorylation were significantly higher than those of the high-glucose intervention group. Following addition of the ERK inhibitor U0126 and the p38 inhibitor SB203580, the levels of ERK and p38 protein phosphorylation declined, as did cell proliferation. These results indicate that irisin promotes β cell proliferation through the ERK and p38 MAPK signaling pathways. Previous studies have reported that irisin can promote the proliferation of human umbilical vein endothelial cells by activating the ERK signaling pathway while having no effect on the p38 MAPK signaling pathway[3]. This may be related to the difference in cell lines used. Since no irisin receptor has yet been identified, the MAPK signaling pathway may be one of the major pathways through which irisin acts.

Previous studies have shown that irisin could inhibit the high-glucose-induced apoptosis of endothelial cells[3]. Experiments in vitro indicated that irisin can increase the expression of antioxidant enzymes to inhibit oxidative stress and high-glucose-induced apoptosis of vascular endothelial cells[5]. Irisin can also decrease the oxidative stress induced by high sugar/high fat in human umbilical vein endothelial cells by inhibiting the activation of the PKC-β/NADPH oxidase and NF-κB/iNOS signaling pathways. In other words, irisin can inhibit cell apoptosis and improve the function of vascular endothelial cells[13]. Thus, this study explored whether irisin could inhibit high-glucose-induced apoptosis of pancreatic β cells. We demonstrated that the number of apoptotic cells was increased significantly after intervention with high glucose compared with that of the control group. INS-1 cell apoptosis was significantly reduced in the irisin intervention group compared with that in the high-glucose group. Our results show that irisin can inhibit the apoptosis of pancreatic β cells induced by high glucose.

Next, we explored whether irisin can inhibit the apoptosis of pancreatic β cells by regulating caspase family proteins, which are involved in apoptosis. Western blot results showed that the levels of pro-apoptotic proteins increased significantly and the levels of anti-apoptotic proteins decreased after the intervention with high glucose. After addition of irisin, the levels of pro-apoptotic proteins, such as cleaved caspase-3, cleaved caspase-9, Bad and Bax were down-regulated, and the levels of anti-apoptotic proteins, such as Bcl-2 and Bcl-xl, were up-regulated. These results demonstrated that irisin could inhibit the apoptosis of β cells by regulating the levels of proteins that are involved in apoptotic pathways. This finding is consistent with previous studies showing that irisin inhibited the high-glucose-induced apoptosis of endothelial cells by up-regulating the expression of the anti-apoptotic protein Bcl-2 and down-regulating the expression of the pro-apoptotic proteins cleaved caspase-3, cleaved caspase-9 and Bax[3].

High-glucose toxicity can damage the secretory function of pancreatic β cells. This study also examined whether irisin can improve the function of pancreatic β cells under hyperglycemic conditions. Our results showed that irisin enhanced glucose-stimulated insulin secretion by INS-1 cells when the cells were treated with irisin and high glucose. Moreover, insulin secretion was significantly reduced when INS-1 cells were treated with high glucose alone.

In summary, we demonstrated that irisin promotes β cell proliferation through the ERK and p38 MAPK signaling pathways. Irisin can also inhibit the high-glucose-induced apoptosis of INS-1 cells and improve the function of pancreatic β cells. Studies are currently underway to further investigate the effects of irisin on pancreatic β cell proliferation and high-glucose-induced apoptosis and to examine the underlying mechanisms.
